# Contrast-Enhanced CT May Be a Diagnostic Alternative for Gastroesophageal Varices in Cirrhosis with and without Previous Endoscopic Variceal Therapy

**DOI:** 10.1155/2019/6704673

**Published:** 2019-10-20

**Authors:** Qianqian Li, Ran Wang, Xiaozhong Guo, Hongyu Li, Xiaodong Shao, Kexin Zheng, Xiaolong Qi, Yingying Li, Xingshun Qi

**Affiliations:** ^1^Department of Gastroenterology, General Hospital of Northern Theater Command, Shenyang 110840, China; ^2^Postgraduate College, Dalian Medical University, Dalian 116044, China; ^3^Postgraduate College, Jinzhou Medical University, Jinzhou 121001, China; ^4^CHESS Group, Hepatic Hemodynamic Lab, Institute of Hepatology, Nanfang Hospital, Southern Medical University, Guangzhou, China

## Abstract

**Background and Aims:**

Liver fibrosis blood tests, platelet count/spleen diameter ratio (PSR), and contrast-enhanced CT are diagnostic alternatives for gastroesophageal varices, but they have heterogeneous diagnostic performance among different study populations. Our study is aimed at evaluating their diagnostic accuracy for esophageal varices (EVs) and gastric varices (GVs) in cirrhotic patients with and without previous endoscopic variceal therapy.

**Methods:**

Patients with liver cirrhosis who underwent blood tests and contrast-enhanced CT scans as well as endoscopic surveillance should be potentially eligible. EVs needing treatment (EVNTs) and GVs needing treatment (GVNTs) were recorded according to the endoscopic results. Area under the curves (AUCs) were calculated.

**Results:**

Overall, 279 patients were included. In 175 patients without previous endoscopic variceal therapy, including primary prophylaxis population (*n* = 70), acute bleeding population (*n* = 38), and previous bleeding population (*n* = 67), the diagnostic accuracy of contrast-enhanced CT for EVNTs was higher (AUCs = 0.816‐0.876) as compared to blood tests and PSR; by comparison, the diagnostic accuracy of contrast-enhanced CT for GVNTs was statistically significant among primary prophylaxis population (AUC = 0.731, *P* = 0.0316), but not acute or previous bleeding population. In 104 patients with previous endoscopic variceal therapy (i.e., secondary prophylaxis population), contrast-enhanced CT was the only statistically significant alternative for diagnosing EVNTs and GVNTs but with modest accuracy (AUCs = 0.673 and 0.661, respectively).

**Conclusions:**

Contrast-enhanced CT might be a diagnostic alternative for EVNTs in cirrhotic patients, but its diagnostic performance was slightly weakened in secondary prophylaxis population. Additionally, contrast-enhanced CT may be considered for diagnosis of GVNTs in primary prophylaxis population without previous endoscopic variceal therapy and secondary prophylaxis population.

## 1. Introduction

Cirrhosis is the end stage of chronic liver disease, which is histologically characterized by fibrosis, scar, and regenerative nodules leading to structural deformation [1]. A major consequence of advanced cirrhosis is portal hypertension, which leads to the development of gastroesophageal varices (GEVs) [2]. Endoscopy should be performed at the time of first diagnosis of liver cirrhosis [3]. GEVs are observed in about 50% of patients with cirrhosis, and 8% of patients without GEVs develop them each year. Patients with no or small varices and without prior history of variceal bleeding should undergo endoscopic surveillance every 1-2 years. Bleeding from GEVs results in a mortality of 5-20% at 6 weeks. Endoscopic treatment, such as endoscopic variceal ligation (EVL) or tissue adhesive injection, is recommended for the management of high-risk varices and acute variceal bleeding [3–5]. However, patients undergoing endoscopic treatment for variceal bleeding have a high variceal recurrence rate of 8-48% [6, 7], a rebleeding rate of 20-43%, and a bleeding related mortality of 19-34% [8]. Therefore, after endoscopic treatment, repeated EVL should be performed every 1-2 weeks until variceal obliteration. The first endoscopic surveillance for variceal recurrence should be performed within 1-3 months after variceal obliteration, and then endoscopic surveillance should be repeated every 6-12 months [5].

Despite endoscopy is the golden approach for diagnosis and surveillance of GEVs according to the current practice guideline and consensus, it is often limited by increased invasiveness, patients' discomfort and poor adherence, and high cost [9–11]. Recently, noninvasive blood tests have been used to diagnose GEVs [12, 13], such as aspartate aminotransferase (AST) to platelet (PLT) ratio index (APRI), AST to alanine aminotransferase (ALT) ratio (AAR), fibrosis 4 index (FIB-4), Lok score, and King score. Contrast-enhanced computed tomography (CT), a conventional diagnostic imaging tool in patients with liver diseases, has also been explored for the assessment of GEVs [14–17]. Additionally, a combination of blood tests with imaging examination for screening GEVs, such as PLT count to spleen diameter ratio (PSR), has been frequently explored [18].

Notably, the performance of these diagnostic alternatives may be heterogeneous among different study populations. However, until now, no study has evaluated their diagnostic accuracy according to the patient characteristics [11]. For this reason, we conducted a retrospective observational study to evaluate the accuracy of blood tests, PSR, and contrast-enhanced CT for diagnosing esophageal varices (EVs) and gastric varices (GVs) in cirrhotic patients with and without variceal bleeding or previous endoscopic variceal therapy.

## 2. Methods

### 2.1. Patients

This was a single-center retrospective observational study on the basis of our prospective database regarding cirrhotic patients undergoing both contrast-enhanced CT and upper gastrointestinal endoscopy. This study was approved by the medical ethical committee of our hospital and the approval number was [k (2018) 08]. The patients' informed consents were waived. All patients consecutively admitted to our department from December 2014 to October 2018 were potentially eligible.

The inclusion criteria were as follows: (1) patients had a diagnosis of liver cirrhosis according to the medical history, clinical features, imaging, and/or histological results and (2) both contrast-enhanced CT and endoscopic examinations were performed at their admissions, and the time interval between the two examinations was within one month. Repeated admission was not excluded.

The exclusion criteria were as follows: (1) patients had a definite diagnosis of malignant tumors, (2) contrast-enhanced CT was performed after endoscopic treatment at their admissions, and (3) contrast-enhanced CT images were not well preserved.

### 2.2. Groups

According to the previous history of endoscopic treatment for variceal bleeding, history of gastrointestinal bleeding (GIB), and presence of acute upper gastrointestinal bleeding (AUGIB), the patients were divided into four groups:

1. *Primary prophylaxis population* (no history of endoscopic treatment, no history of GIB, and absence of AUGIB)
2. *Acute bleeding population* (no history of endoscopic treatment, but with presence of AUGIB, regardless of history of GIB)
3. *Previous bleeding population* (no history of endoscopic treatment, absence of AUGIB, but with a history of GIB)
4. *Secondary prophylaxis population* (a history of endoscopic treatment for variceal bleeding, but absence of GIB)

As for the *secondary prophylaxis population*, the patients would be further excluded, if the time interval between prior endoscopic treatment and present admission was less than one month [19]. This is primarily because the esophagus and stomach lumen mucosa may not be fully recovered during a short postoperative period, which will cause a potential radiological artifact on CT images and influence its diagnostic performance.

### 2.3. Data Collection

The data were collected as follows: age, sex, etiology of liver diseases, ascites, interval between prior endoscopic treatment and present admission, red blood cell (RBC), hemoglobin (Hb), white blood cell (WBC), PLT, total bilirubin (TBIL), direct bilirubin (DBIL), albumin (ALB), ALT, AST, alkaline phosphatase (AKP), *γ*-glutamine transferase (GGT), blood urea nitrogen (BUN), serum creatinine (SCr), prothrombin time (PT), activated partial thromboplastin time (APTT), and international normalized ratio (INR). The maximum diameter of the spleen was measured on axial contrast-enhanced CT images. The Child-Pugh [20] model for end-stage of liver disease (MELD) [21], APRI [22], AAR [23], FIB-4 [24], Lok [25], King [26], and PSR [27] scores were calculated as follows:

(1) $$\begin{array}{c} \text{Child}-\text{Pugh score}=\text{ALB score}+\text{TBIL score}+\text{INR score}+\text{ascites score}+\text{hepatic encephalopathy score},\\ \text{MELD score}=9.57\times \ln\left[\text{Cr }\left(\mu \text{mol}/\text{L}\right)\times 0.011\right]+3.78\times \ln\left[\text{TBIL }\left(\mu \text{mol}/\text{L}\right)\times 0.058\right]+11.2\times \ln\left(\text{INR}\right)+6.43,\\ \text{APRI}=\frac{\left(\text{AST}/\text{upper limit of normal}\right)\times 100}{\text{PLT}},\\ \text{AAR}=\frac{\text{AST}}{\text{ALT}},\\ \text{FIB}‐4=\frac{\text{age}\times \text{AST}}{\text{PLT}\times \text{AL}{\text{T}}^{\left(1/2\right)}},\\ \text{King}=\frac{\text{age}\times \text{AST}\times \text{INR}}{\text{PLT}},\\ \text{logodds}=-5.56-0.0089\times \text{PLT}+1.26\times \left(\frac{\text{AST}}{\text{ALT ratio}}\right)+5.27\times \text{INR},\\ \text{Lok}=\frac{\exp\;\left(\text{logodds}\right)}{1+\exp\;\left(\text{logodds}\right)},\\ \text{PSR}=\frac{\text{PLT}}{\text{spleen diameter}}. \end{array}$$

### 2.4. Contrast-Enhanced CT Images

Two observers (QL and RW) used the patients' names or case numbers to search contrast-enhanced CT images in the PowerRIS system. Notably, they were blinded to the laboratory and endoscopic findings when the CT images were retrospectively analyzed. They independently evaluated the presence of GEVs. EVs or GVs were defined as enhancing lesions abutted the luminal surface of the esophageal or gastric wall or protruded into esophageal or gastric luminal space at the portal vein phases of contrast-enhanced CT images [28, 29]. They also independently selected the CT layer with the maximum diameter of varices. In cases of any inconsistency in measuring the maximum diameter of varices between the two observers, a discussion with another investigator (XQ) was made until a consensus was achieved. Additionally, they evaluated the spleen and measured the maximum diameter of the spleen on contrast-enhanced CT images.

### 2.5. Endoscopy

In the present study, an endoscopist (DS) underwent all endoscopic examinations. The shape of EVs and red color (RC) signs were described, and then the grade of EVs was evaluated. The grade of EVs is classified into no, mild, moderate, and severe according to the 2008 Hangzhou consensus [30]. The detailed definitions are as follows: (1) mild EVs: straight or slight tortuous EVs without RC signs; (2) moderate EVs: straight or slightly tortuous EVs with RC signs or serpentine tortuous uplifted EVs without RC signs; and (3) severe EVs: serpentine tortuous uplifted EVs with RC signs or beaded, nodular, or tumor-like EVs with or without RC signs. EVs needing treatment (EVNTs) were further defined as moderate and severe EVs. The presence of GVs was also evaluated. GVs needing treatment (GVNTs) were further defined as large GVs or RC signs in the GVs at the discretion of our endoscopist.

### 2.6. Statistical Analysis

All statistical analyses were performed using the SPSS software version 20.0 (IBM Corp, Armonk, NY, USA) and MedCalc software version 11.4.2.0 (MedCalc Software, Mariakerke, Belgium). Data were expressed as mean ± standard deviation, median and range, or frequencies and percentages. Kappa statistics were used to explore the agreement of diagnosing presence of EVs and GVs between two observers. Receiver operating characteristic (ROC) curve was used to explore the diagnostic performance of blood tests, PSR, and contrast-enhanced CT. We calculated the area under the curve (AUC) and compared them by using the DeLong test. *P* < 0.05 was considered statistically significant. Additionally, we determined the optimal cutoff values of contrast-enhanced CT by reaching the maximal negative predictive value (NPV) and then calculated the rates of spared endoscopy and missed varices. The bar charts were drawn by the Excel version 16.0 (Microsoft Corp, Redmond, Washington, USA).

## 3. Results

### 3.1. Patients

**Figure 1:**
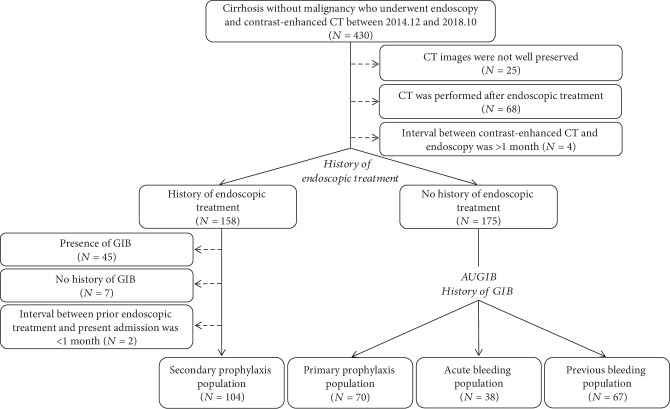
Flow chart of patient enrollment. CT: computed tomography; AUGIB: acute upper gastrointestinal bleeding.

A total of 430 cirrhotic patients underwent both contrast-enhanced CT and endoscopic examinations. Finally, a total of 279 cirrhotic patients were included (Figure 1). Baseline characteristics are shown in Table 1. Results of kappa statistics were shown in Supplementary [Supplementary-material supplementary-material-1].

### 3.2. Primary Prophylaxis Population

Seventy patients were included in this group. Prevalence of EVs, EVNTs, GVs, and GVNTs was 61.4% (43/70), 37.1% (26/70), 25.7% (18/70), and 11.4% (8/70), respectively. As for EVs, only contrast-enhanced CT, Lok score, and PSR had statistically significant diagnostic performance; as for EVNTs, only contrast-enhanced CT and PSR had statistically significant diagnostic performance; as for GVs, only contrast-enhanced CT, AAR score, Lok score, and PSR had statistically significant diagnostic performance; as for GVNTs, only contrast-enhanced CT had statistically significant diagnostic performance (Table 2).

**Figure 2:**
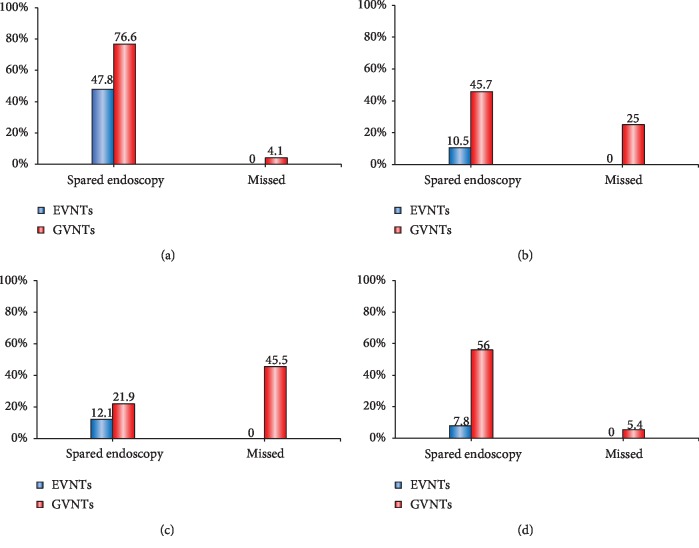
Bar charts showing the rates of spared endoscopy and missed varices by contrast-enhanced CT for predicting the presence of EVNTs and GVNTs in different population. (a) Performance in *primary prophylaxis population*. (b) Performance in *acute bleeding population*. (c) Performance in *previous bleeding population*. (d) Performance in *secondary prophylaxis population*. CT: computed tomography; EVNTs: esophageal varices needing treatment; GVNTs: gastric varices needing treatment.

The presence of EVs and diameter of EVs could be evaluated on CT in all of the 70 patients. The diameter of EVs measured on contrast-enhanced CT < 0.50 cm should be considered as the optimal cutoff value for ruling out the EVNTs. By using this cutoff value, 47.8% (32/67) of endoscopies were spared, and no (0/32) EVNTs was missed (Figure 2(a)).

After a discussion among investigators, the presence of GVs could not be evaluated on CT in one patient and the diameter of GVs could not be measured on CT in 3 patients. The diameter of GVs measured on contrast-enhanced CT < 1.09 cm should be considered as the optimal cutoff value for ruling out the GVNTs. By using this cutoff value, 76.6% (49/64) of endoscopies were spared, but 4.1% (2/49) of GVNTs were missed (Figure 2(a)).

### 3.3. Acute Bleeding Population

Thirty-eight patients were included in this group. Prevalence of EVs, EVNTs, GVs, and GVNTs was 92.1% (35/38), 71.1% (27/38), 50.0% (19/38), and 39.5% (15/38), respectively. As for EVs, contrast-enhanced CT, APRI score, FIB-4 score, King score, Lok score, and PSR had statistically significant diagnostic performance; as for EVNTs, only contrast-enhanced CT and PSR had statistically significant diagnostic performance; as for GVs and GVNTs, all alternatives did not have any statistically significant diagnostic performance (Table 2).

The presence of EVs and diameter of EVs could be evaluated on CT in all of the 38 patients. The diameter of EVs measured on contrast-enhanced CT < 0.38 cm should be considered as the optimal cutoff value for ruling out the EVNTs. By using this cutoff value, 10.5% (4/38) of endoscopies were spared, and no (0/4) EVNTs was missed (Figure 2(b)).

After a discussion among investigators, the diameter of GVs could not be measured on CT in 3 patients. The diameter of GVs measured on contrast-enhanced CT < 1.01 cm should be considered as the optimal cutoff value for ruling out the GVNTs. By using this cutoff value, 45.7% (16/35) of endoscopies were spared, but 25% (4/16) of GVNTs were missed (Figure 2(b)).

### 3.4. Previous Bleeding Population

Sixty-seven patients were included in this group. Prevalence of EVs, EVNTs, GVs, and GVNTs was 91.0% (61/67), 73.1% (49/67), 73.1% (49/67), and 53.7% (36/67), respectively. As for EVs, only contrast-enhanced CT had statistically significant diagnostic performance; as for EVNTs, only contrast-enhanced CT and PSR had statistically significant diagnostic performance; as for GVs, only contrast-enhanced CT had statistically significant diagnostic performance; as for GVNTs, only AAR score had statistically significant diagnostic performance (Table 2).

The presence of EVs and diameter of EVs could be evaluated on CT in all of the 67 patients. The diameter of EVs measured on contrast-enhanced CT < 0.46 cm should be considered as the optimal cutoff value for ruling out the EVNTs. By using this cutoff value, 12.1% (8/66) of endoscopies were spared, and no (0/8) EVNTs was missed (Figure 2(c)).

After a discussion among investigators, the diameter of GVs could not be measured on CT in 3 patients (3/67). The diameter of GVs measured on contrast-enhanced CT < 0.95 cm should be considered as the optimal cutoff value for ruling out the GVNTs. By using this cutoff value, 21.9% (14/64) of endoscopies were spared, but 45.5% (5/14) of GVNTs were missed (Figure 2(c)).

### 3.5. Secondary Prophylaxis Population

One hundred and four patients were included in this group. Prevalence of EVs, EVNTs, GVs, and GVNTs was 90.4% (94/104), 40.4% (42/104), 34.6% (36/104), and 15.4% (16/104), respectively.

As for EVs, only contrast-enhanced CT had statistically significant diagnostic performance; as for EVNTs, only contrast-enhanced CT and AAR score had statistically significant diagnostic performance; as for GVs, only contrast-enhanced CT had statistically significant diagnostic performance; as for GVNTs, only contrast-enhanced CT and FIB-4 score had statistically significant diagnostic performance (Table 2).

After a discussion among investigators, the diameter of EVs could not be measured on CT in one patient. The diameter of EVs measured on contrast-enhanced CT < 0.33 cm should be considered as the optimal cutoff value for ruling out the EVNTs. By using this cutoff value, 7.8% (8/103) of endoscopies were spared, and no (0/8) EVNTs was missed (Figure 2(d)).

After a discussion among investigators, the presence of GVs could not be evaluated on CT in 2 patients and the diameter of GVs could not be measured on CT in 2 patients. The diameter of GVs measured on contrast-enhanced CT < 1.11 cm should be considered as the optimal cutoff value for ruling out the GVNTs. By using this cutoff value, 56% (56/100) of endoscopies were spared, but 5.4% (3/56) of GVNTs were missed (Figure 2(d)).

## 4. Discussion

Currently, noninvasive diagnosis of GEVs is a hot topic. Severity of liver fibrosis is often in parallel with that of portal hypertension in compensated cirrhosis. Thus, the markers reflecting the severity of liver fibrosis are frequently used for noninvasive assessment of portal hypertension in such patients [10, 31]. Considering that liver stiffness measured by transient elastography can stage liver fibrosis and PLT indicates portal hypertension, Baveno VI consensus has recommended that liver stiffness < 20 kPa combined with PLT > 150 × 10^9^/L should be a criterion for sparing endoscopy in compensated cirrhosis [4], and only a minority of patients within this Baveno VI criterion have a risk of variceal bleeding [32]. Researchers attempted to further improve its diagnostic accuracy by means of optimizing the thresholds of liver stiffness and PLT or establishing stepwise ruling-out and/or ruling-in strategies (Supplementary [Supplementary-material supplementary-material-1]). Noninvasive approaches on the basis of Baveno VI criterion can accurately diagnose EVNTs with a missing rate of <5% [33–43]. Despite so, it should be noted that Baveno VI criterion should be appropriate for only patients with compensated cirrhosis without any history of gastrointestinal bleeding or endoscopic treatment. By comparison, few well-established tools have been employed for patients with advanced and decompensated cirrhosis, in whom extrahepatic factors, such as development of extrahepatic collaterals and splanchnic vasodilation, became more important for the progression of portal hypertension than intrahepatic resistance caused by liver fibrosis [44]. In this setting, we have for the first time evaluated the diagnostic accuracy of blood tests, PSR, and contrast-enhanced CT for GEVs according to the severity of liver cirrhosis and portal hypertension, including patients without variceal bleeding (*primary prophylaxis population*), with variceal bleeding (*acute bleeding population* and *previous bleeding population*), and with history of endoscopic treatment for variceal bleeding (*secondary prophylaxis population*).

Our previous meta-analysis demonstrated that APRI, AAR, FIB-4, and Lok scores had low to moderate diagnostic accuracy in predicting the presence of EVs and EVNTs in liver cirrhosis, and their AUCs were 0.6774-0.7885 and 0.7095-0.7448, respectively [12]. Notably, among the studies included in the meta-analysis, most of patients had well-preserved liver function. By comparison, our previous observational study where a majority of patients were decompensated demonstrated that APRI, AAR, FIB-4, and Lok scores had low accuracy for EVs and EVNTs with AUCs of 0.539-0.567 and 0.506-0.544, respectively [13]. Similarly, our present observational study also confirmed that these blood tests were insufficient to replace endoscopy in diagnosing EVs, EVNTs, GVs, and GVNTs in advanced decompensated patients.

PSR had relatively high diagnostic accuracy in predicting the presence of EVs in compensated cirrhotic patients and its AUC was 0.85 [18]. The advantages of PSR as a potential diagnostic alternative for EVs can be explained by the fact that splenomegaly and hypersplenism are common clinical manifestations of portal hypertension, and the PSR model associates decreased PLT with splenomegaly [27, 45]. By contrast, our present study suggested that PSR was unsatisfactory for prediction of GEVs. This might be related to the characteristics of our patients that a majority of patients in *primary prophylaxis population* group had Child-Pugh class B or C and all patients in 3 other groups (i.e., *secondary prophylaxis population*, *acute bleeding population*, and *previous bleeding population*) were decompensated with recent or previous bleeding. This was in consistency with the results of a previous study which also included patients receiving secondary prophylaxis and achieved only an AUC of 0.715 [46].

Our previous meta-analysis demonstrated that contrast-enhanced CT had high diagnostic accuracy in predicting the presence of EVs, EVNTs, and GVs, and their AUCs were 0.8958, 0.9461, and 0.9127, respectively [14]. Similarly, another meta-analysis also confirmed that the AUCs were 0.86 and 0.95 in predicting the presence of EVs and GVs, respectively [15]. By comparison, our present study confirmed such high diagnostic accuracy of contrast-enhanced CT in predicting EVs and EVNTs and further suggested that no EVNTs would be missed according to the optimal cutoff value. However, the diagnostic performance of contrast-enhanced CT was insufficient in *secondary prophylaxis population*.

**Figure 3:**
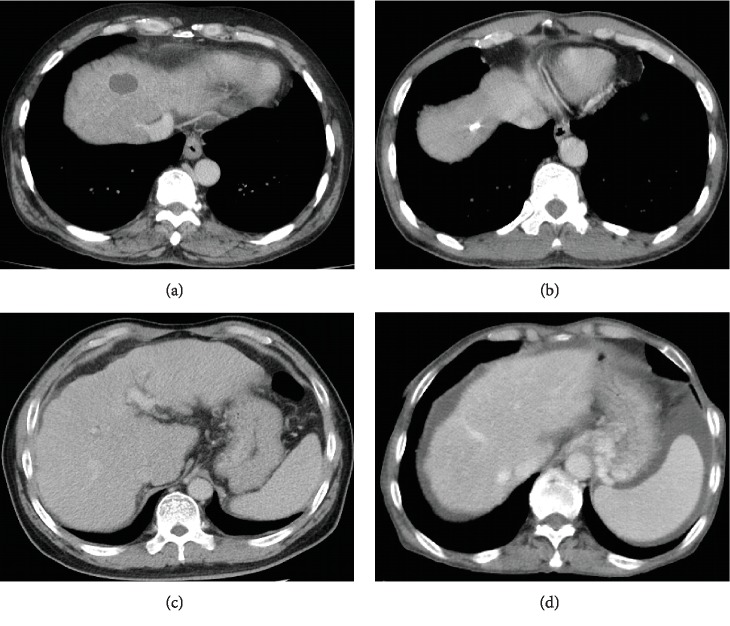
Pitfalls in diagnosis of GEVs on contrast-enhanced CT. (a) Esophageal wall became stiff after repeated endoscopic treatments. (b) Small EVs were observed on contrast-enhanced CT, but missed on endoscopy. (c) GVs could not be evaluated as gastric cavity was not fully expanded. (d) GVs appeared as irregular vascular shadows, where the maximum diameter of varices was hard to be measured. CT: computed tomography; GEVs: gastroesophageal varices; EVs: esophageal varices; GVs: gastric varices.

Several pitfalls of contrast-enhanced CT scans for assessment of GEVs should be recognized. First, esophageal wall may form scars and stiffen after repeated endoscopic treatments, in which enhanced vascular shadows do not obviously protrude into esophageal lumen on contrast-enhanced CT images (Figure 3(a)). Second, during the endoscopic examinations, small EVs may be flattened after dilating esophageal lumen, thereby leading to a missed diagnosis (Figure 3(b)). Third, the images obtained at the portal vein phases of contrast-enhanced CT scans are inappropriately selected by radiological technicians, in which esophageal venous vessels cannot be obviously enhanced. Fourth, abdominal CT scans are selected for our present study, in which the lesions at middle and upper esophagus cannot be observed. Fifth, contrast-enhanced CT scans can detect GVs located deeply in gastric mucosa [29], which are hard to be distinguished from gastric mucosal folds by endoscopy. Sixth, when the gastric cavity is not fully expanded, small GVs do not protrude from the surface and cannot be differentiated from the gastric mucosa folds on CT images (Figure 3(c)). Seventh, some GVs appear as irregular vascular shadows on contrast-enhanced CT images, thereby misjudging the maximum diameter of varices (Figure 3(d)).

Several other advantages of contrast-enhanced CT scans should not be ignored, because it can simultaneously evaluate the severity of liver cirrhosis and its related complications, such as grade or quantification of ascites [47], thrombosis within portal vein system [48], portosystemic collaterals [49], and liver cancer [50], except for GEVs. On the other hand, the disadvantages of contrast-enhanced CT scans include the following. First, the risk of radiation will be increased. Second, contrast-enhanced CT is not applicable to patients with renal failure, hyperthyroidism, and hypersensitivity to contrast media. Third, RC sign is valuable for evaluating the severity of GEVs, but it cannot be observed on contrast-enhanced CT images.

**Figure 4:**
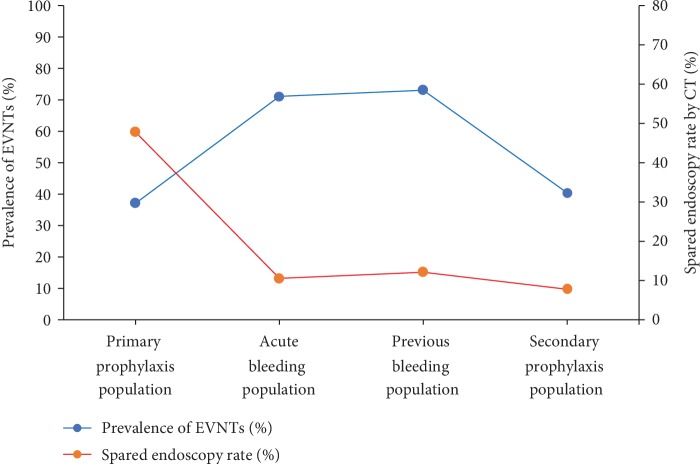
Line chart showing the relation between the rates of spared endoscopy and prevalence of EVNTs in different populations. EVNTs: esophageal varices needing treatment.

Our study had several limitations. First, Western studies evaluated EVNTs by the size of EVs under endoscopy, and our study employed the Chinese guideline to identify EVNTs. Second, our patients were more severe and had a high prevalence of EVNTs. Because the prevalence of EVNTs should be inversely associated with the rate of spared endoscopy, the rate of sparing more endoscopy was relatively lower in our study (Figure 4). Third, the present study was of the retrospective nature and performed at a single center. Fourth, the sample size was small in different study population, especially in *acute bleeding population*.

In conclusion, contrast-enhanced CT seemed to have higher diagnostic accuracy for EVs and EVNTs in cirrhotic patients as compared to APRI, AAR, FIB-4, FI, Lok, and King scores and PSR. Among the *secondary prophylaxis population* requiring repeated endoscopic surveillance, contrast-enhanced CT seemed to be the only useful diagnostic alternative for GEVs in cirrhotic patients. However, the potential pitfalls of contrast-enhanced CT, such as stiff and scarred esophagus, small or irregular vascular shadows, and technical errors, can decrease its diagnostic accuracy in *secondary prophylaxis population*.

## Figures and Tables

**Table 1:** Baseline characteristics of patients.

Variables	Primary prophylaxis population		Acute bleeding population		Previous bleeding population		Secondary prophylaxis population	
	No. pts	Mean ± SD, median (range), or frequency (percentage)	No. pts	Mean ± SD, median (range), or frequency (percentage)	No. pts	Mean ± SD, median (range), or frequency (percentage)	No. pts	Mean ± SD, median (range), or frequency (percentage)
Age (years)	70	56.67 ± 9.77 57.61 (26.74-78.64)	38	53.32 ± 12.52 52.72 (20.58-80.79)	67	53.11 ± 10.01 50.56 (33.30-78.94)	104	57.10 ± 11.37 58.31 (20.87-79.07)
Sex (male)	70	51 (72.9%)	38	32 (84.2%)	67	50 (74.6%)	104	77 (74.0%)
Etiology of liver diseases								
HBV infection	70	28 (40.0%)	38	13 (34.2%)	67	23 (34.3%)	104	46 (44.2%)
HCV infection	70	4 (5.7%)	38	2 (5.3%)	67	9 (13.4%)	104	9 (8.7%)
Alcohol abuse	70	30 (42.9%)	38	17 (44.7%)	67	29 (43.3%)	104	37 (35.6%)
Drug related	70	8 (11.4%)	38	3 (7.9%)	67	8 (11.9%)	104	7 (6.7%)
Autoimmune related	70	3 (4.3%)	38	1 (2.6%)	67	3 (4.5%)	104	7 (6.7%)
Ascites	70		38		67		104	
No		33 (47.1%)		14 (36.8%)		32 (47.8%)		42 (40.4%)
Mild		11 (15.7%)		14 (36.8%)		18 (26.9%)		40 (38.5%)
Moderate-severe		26 (37.1%)		10 (26.3%)		17 (25.4%)		22 (21.2%)
Interval between prior endoscopic treatment and present admission (years)							100^a^	0.93 ± 0.99 0.61 (0.10-5.78)
Interval between CT and endoscopy (days)	70	4.96 ± 3.85 4.00 (0.00-18.00)	38	2.50 ± 2.05 2.00 (1.00-9.00)	67	3.11 ± 2.46 3.00 (0.00-17.00)	104	2.56 ± 2.45 2.00 (0.00-15.00)
RBC (10^12^/L)	70	3.78 ± 0.68 3.88 (1.45-5.06)	38	2.73 ± 0.80 2.58 (1.51-5.08)	67	3.17 ± 0.82 3.22 (1.15-5.05)	104	4.00 ± 0.63 4.05 (1.82-5.49)
Hb (g/L)	70	121.26 ± 22.19 124.00 (55.00-159.00)	38	80.16 ± 26.07 75.50 (37.00-156.00)	67	85.97 ± 25.91 86.00 (28.00-154.00)	104	108.78 ± 22.36 110.50 (33.00-161.00)
WBC (10^9^/L)	70	4.83 ± 2.77 4.00 (1.80-20.80)	38	5.15 ± 4.15 4.25 (1.10-22.40)	67	3.76 ± 2.86 3.20 (0.80-20.30)	104	3.70 ± 2.16 3.40 (0.80-16.70)
PLT (10^9^/L)	70	103.57 ± 74.90 80.00 (22.00-423.00)	38	82.39 ± 39.11 78.00 (26.00-162.00)	67	95.71 ± 68.05 76.00 (23.00-316.00)	104	111.91 ± 77.51 89.50 (23.00-448.00)
TBIL (*μ*mol/L)	70	47.34 ± 42.16 31.05 (6.60-216.50)	38	26.93 ± 20.95 21.90 (5.20-119.30)	67	27.11 ± 29.23 20.00 (5.50-215.30)	104	21.59 ± 13.10 18.30 (5.90-92.60)
DBIL (*μ*mol/L)	70	25.23 ± 27.11 14.30 (2.00-149.90)	38	13.86 ± 14.12 10.15 (2.00-81.80)	67	14.60 ± 23.84 8.90 (2.30-179.30)	104	8.94 ± 5.88 7.65 (2.10-48.90)
ALB (g/L)	69	32.44 ± 7.13 30.30 (19.20-50.60)	38	29.85 ± 5.92 30.10 (19.00-45.40)	67	32.95 ± 6.47 33.60 (14.20-45.30)	103	35.71 ± 4.76 35.90 (22.90-45.60)
ALT (U/L)	70	60.66 ± 73.48 36.72 (7.53-429.98)	38	40.08 ± 33.96 26.21 (9.59-152.11)	67	28.25 ± 18.58 23.09 (4.47-99.13)	104	24.75 ± 12.09 21.07 (9.62-86.13)
AST (U/L)	70	75.95 ± 71.95 60.65 (13.94-394.45)	38	54.38 ± 42.90 39.16 (10.99-202.40)	67	41.76 ± 27.65 32.88 (13.83-151.35)	104	33.03 ± 12.55 30.30 (16.26-70.37)
AKP (U/L)	70	139.04 ± 76.06 113.19 (33.00-400.01)	38	99.51 ± 49.88 84.54 (31.00-232.70)	67	112.09 ± 65.98 86.27 (40.65-399.34)	104	112.54 ± 62.90 97.65 (30.04-466.34)
GGT (U/L)	70	171.27 ± 303.69 73.83 (10.93-1779.18)	38	138.88 ± 241.13 49.57 (12.00-1227.00)	67	74.81 ± 87.66 34.10 (8.23-392.55)	104	64.32 ± 166.61 32.50 (10.50-1680-03)
BUN (mmol/L)	70	10.01 ± 40.54 4.98 (0.64-344.00)	38	8.14 ± 7.71 5.72 (1.86-47.25)	67	5.09 ± 1.76 4.79 (1.57-9.38)	103	5.37 ± 1.95 5.12 (2.28-17.82)
SCr (*μ*mol/L)	70	66.45 ± 20.42 64.65 (23.83-121.45)	38	75.14 ± 36.11 72.62 (32.65-267.63)	67	63.39 ± 15.67 59.04 (37.66-114.13)	103	65.42 ± 16.68 62.97 (36.39-141.50)
PT (seconds)	68	16.27 ± 2.95 15.40 (11.20-28.00)	38	16.76 ± 3.59 15.95 (11.60-27.20)	67	16.48 ± 2.63 16.40 (10.40-25.70)	102	15.64 ± 2.19 15.20 (11.00-25.20)
APTT (seconds)	68	41.64 ± 6.70 40.75 (28.00-64.80)	38	40.03 ± 4.71 39.60 (30.80-51.00)	67	40.72 ± 5.51 40.10 (26.70-52.80)	102	40.37 ± 5.23 39.80 (28.10-60.50)
INR	68	1.32 ± 0.31 1.25 (0.95-2.77)	38	1.40 ± 0.37 1.31 (1.01-2.51)	67	1.35 ± 0.26 1.33 (0.90-2.39)	102	1.26 ± 0.22 1.22 (0.96-2.41)
Child-Pugh class	67^b^		38		67		102^b^	
A		20 (29.9%)		11 (28.9%)		38 (56.7%)		54 (52.9%)
B		32 (47.8%)		21 (55.3%)		23 (34.3%)		47 (46.1%)
C		15 (22.4%)		6 (15.8%)		6 (9.0%)		1 (1.0%)
Child-Pugh score	67^b^	7.79 ± 2.16 8.00 (5.00-13.00)	38	7.68 ± 1.82 8.00 (5.00-12.00)	67	6.90 ± 1.78 6.00 (5.00-12.00)	102^b^	6.50 ± 1.30 6.00 (5.00-10.00)
MELD score	68^c^	8.60 ± 6.06 7.49 (-3.03-27.42)	38	8.28 ± 4.69 7.83 (-3.16-16.73)	67	6.66 ± 4.59 6.35 (-2.73-24.73)	102^c^	5.80 ± 3.79 5.30 (-1.75-19.12)
Spleen diameter (mm)	68^d^	128.83 ± 27.63 126.10 (59.80-190.30)	37^d^	135.96 ± 27.30 134.10 (66.60-189.70)	60^d^	142.05 ± 25.59 143.55 (79.10-189.00)	81^d^	147.97 ± 33.13 147.40 (80.40-248.00)
PSR	68^d^	894.15 ± 786.97 592.19 (177.99-3361.20)	37^d^	641.10 ± 380.74 567.40 (148.57-1654.75)	60^d^	629.77 ± 483.09 458.57 (159.50-2703.67)	81^d^	626.04 ± 451.47 481.48 (121.95-2835.82)
APRI score	70	2.56 ± 2.29 1.87 (0.10-12.03)	38	1.99 ± 1.70 1.74 (0.31-7.67)	67	1.50 ± 1.20 1.33 (0.12-6.10)	104	1.06 ± 0.67 0.90 (0.11-3.44)
AAR score	70	1.55 ± 0.79 1.43 (0.49-5.06)	38	1.49 ± 0.74 1.31 (0.47-3.94)	67	1.65 ± 0.84 1.50 (0.44-5.41)	104	1.45 ± 0.46 1.38 (0.58-3.28)
FIB-4 score	70	7.73 ± 5.42 6.23 (0.71-22.42)	38	6.51 ± 4.02 6.08 (0.96-20.33)	67	6.08 ± 4.19 5.57 (0.82-21.83)	104	5.13 ± 3.61 4.32 (0.72-17.58)
King score	68^c^	82.65 ± 90.75 56.88 (2.02-495.85)	38	61.56 ± 56.76 39.02 (7.26-219.22)	67	44.50 ± 39.43 31.94 (2.99-217.93)	102^c^	31.63 ± 24.04 24.46 (2.60-126.09)
Lok score	68^c^	0.80 ± 0.22 0.89 (0.23-1.00)	38	0.87 ± 0.14 0.92 (0.39-1.00)	67	0.86 ± 0.18 0.93 (0.16-1.00)	102^c^	0.78 ± 0.22 0.87 (0.13-1.00)
EVs	70		38		67		104	
No		27 (38.6%)		3 (7.9%)		6 (9.0%)		10 (9.6%)
Yes		43 (61.4%)		35 (92.1%)		61 (91.0%)		94 (90.4%)
Unknown		0 (0.0%)		0 (0.0%)		0 (0.0%)		0 (0.0%)
EVNTs	70		38		67		104	
No		41 (58.6%)		11 (28.9%)		17 (25.4%)		62 (59.6%)
Yes		26 (37.1%)		27 (71.1%)		49 (73.1%)		42 (40.4%)
Unknown		3 (4.3%)^e^		0 (0.0%)		1 (1.5%)^e^		0 (0.0%)
GVs	70		38		67		104	
No		51 (72.9%)		19 (50.0%)		18 (26.9%)		68 (65.4%)
Yes		18 (25.7%)		19 (50.0%)		49 (73.1%)		36 (34.6%)
Unknown		1 (1.4%)^e^		0 (0.0%)		0 (0.0%)		0 (0.0%)
GVNTs	70		38		67		104	
No		60 (85.7%)		23 (60.5%)		31 (46.3%)		88 (84.6%)
Yes		8 (11.4%)		15 (39.5%)		36 (53.7%)		16 (15.4%)
Unknown		2 (2.9%)^e^		0 (0.0%)		0 (0.0%)		0 (0.0%)

^a^The specific date of previous endoscopic treatment could not be obtained in 4 patients. ^b^Child-Pugh score could not be evaluated due to the absence of ALB or INR. ^c^MELD, King, and Lok score could not be evaluated due to the absence of INR. ^d^Spleen diameter and PSR were not available in patients with splenectomy. ^e^EVNTs, GVs, and GVNTs could not be evaluated due to the absence of detailed endoscopic reports. SD: standard deviation; HBV: hepatitis B virus; HCV: hepatitis C virus; CT: computed tomography; RBC: red blood cell; Hb: hemoglobin; WBC: white blood cell; PLT: platelet; TBIL: total bilirubin; DBIL: direct bilirubin; ALB: albumin; ALT: alanine aminotransferase; AST: aspartate aminotransferase; AKP: alkaline phosphatase; GGT-*γ*: glutamyl transpeptidase; BUN: blood urea nitrogen; SCr: serum creatinine; PT: prothrombin time; APTT: activated partial thromboplastin time; INR: international normalized ratio; MELD: model for end-stage liver disease; PSR: PLT count to spleen diameter ratio; APRI: AST to PLT ratio index; AAR: AST to ALT ratio; FIB4: fibrosis 4 index; EVs: esophageal varices; EVNTs: esophageal varices needing treatment; GVs: gastric varices; GVNTs: gastric varices needing treatment.

**Table 2:** Diagnostic performance of alternative approaches.

Variables	Primary prophylaxis population			Acute bleeding population			Previous bleeding population			Secondary prophylaxis population		
	No. pts	AUC (95% CI)	*P* value	No. pts	AUC (95% CI)	*P* value	No. pts	AUC (95% CI)	*P* value	No. pts	AUC (95% CI)	*P* value
EVs												
APRI score	70	0.550 (0.426-0.669)	0.5207	38	0.876 (0.729-0.960)	**<0.0001**	67	0.523 (0.398-0.647)	0.8813	104	0.532 (0.432-0.630)	0.7526
AAR score	70	0.550 (0.426-0.669)	0.5142	38	0.714 (0.545-0.849)	0.4083	67	0.672 (0.547-0.782)	0.2117	104	0.513 (0.413-0.613)	0.8961
FIB4 score	70	0.632 (0.508-0.744)	0.0852	38	0.771 (0.607-0.892)	**0.0314**	67	0.538 (0.412-0.661)	0.8163	104	0.536 (0.436-0.635)	0.7489
King score	68	0.586 (0.460-0.704)	0.2556	38	0.838 (0.683-0.937)	**0.0002**	67	0.500 (0.375-0.625)	1.0000	102	0.525 (0.424-0.625)	0.8078
Lok score	68	0.654 (0.529-0.766)	**0.0342**	38	0.905 (0.765-0.976)	**<0.0001**	67	0.503 (0.378-0.627)	0.9863	102	0.593 (0.491-0.689)	0.4019
PSR^∗^	68	0.755 (0.636-0.852)	**0.0001**	37	0.882 (0.734-0.965)	**<0.0001**	60	0.664 (0.530-0.780)	0.2587	81	0.633 (0.519-0.738)	0.2900
Contrast-enhanced CT	70	0.680 (0.588-0.787)	**0.0004**	38	0.833 (0.677-0.934)	**0.0455**	67	0.833 (0.722-0.913)	**0.0016**	104	0.739 (0.644-0.821)	**0.0042**
EVNTs												
APRI score	67	0.490 (0.366-0.615)	0.8912	38	0.513 (0.346-0.679)	0.8984	66	0.551 (0.424-0.674)	0.5592	104	0.564 (0.463-0.661)	0.2649
AAR score	67	0.475 (0.352-0.601)	0.7264	38	0.648 (0.477-0.796)	0.1905	66	0.547 (0.419-0.670)	0.5854	104	0.616 (0.516-0.710)	**0.0344**
FIB4 score	67	0.542 (0.416-0.665)	0.5567	38	0.549 (0.379-0.710)	0.6382	66	0.500 (0.374-0.626)	1.0000	104	0.502 (0.402-0.601)	0.9786
King score	65	0.516 (0.389-0.642)	0.8272	38	0.505 (0.338-0.671)	0.9608	66	0.571 (0.444-0.693)	0.4147	102	0.519 (0.418-0.619)	0.7456
Lok score	65	0.557 (0.428-0.680)	0.4315	38	0.582 (0.412-0.740)	0.4786	66	0.570 (0.442-0.691)	0.4231	102	0.546 (0.444-0.644)	0.4251
PSR^∗^	65	0.670 (0.542-0.782)	**0.0126**	37	0.738 (0.567-0.868)	**0.0127**	59	0.688 (0.554-0.802)	**0.0185**	81	0.595 (0.480-0.703)	0.1428
Contrast-enhanced CT	67	0.876 (0.772-0.944)	**<0.0001**	38	0.816 (0.658-0.923)	**0.0001**	66	0.873 (0.768-0.942)	**<0.0001**	103	0.673 (0.574-0.762)	**0.0012**
GVs												
APRI score	69	0.541 (0.417-0.662)	0.5846	38	0.589 (0.418-0.745)	0.3527	67	0.588 (0.461-0.707)	0.2517	104	0.532 (0.432-0.631)	0.6022
AAR score	69	0.709 (0.587-0.812)	**0.0009**	38	0.611 (0.439-0.764)	0.2412	67	0.549 (0.423-0.671)	0.5593	104	0.565 (0.464-0.662)	0.2948
FIB4 score	69	0.636 (0.512-0.749)	0.0679	38	0.535 (0.366-0.698)	0.7206	67	0.621 (0.494-0.737)	0.1027	104	0.499 (0.399-0.599)	0.9867
King score	67	0.546 (0.420-0.669)	0.5393	38	0.554 (0.384-0.715)	0.5756	67	0.618 (0.491-0.734)	0.1236	104	0.508 (0.407-0.609)	0.8952
Lok score	67	0.672 (0.547-0.782)	**0.0079**	38	0.551 (0.382-0.713)	0.6018	67	0.499 (0.375-0.624)	0.9944	102	0.534 (0.432-0.633)	0.5642
PSR^∗^	67	0.664 (0.538-0.774)	**0.0236**	37	0.614 (0.440-0.769)	0.2334	60	0.603 (0.469-0.727)	0.2093	81	0.510 (0.396-0.623)	0.8834
Contrast-enhanced CT	68	0.721 (0.599-0.823)	**0.0005**	38	0.605 (0.434-0.760)	0.1797	67	0.671 (0.546-0.781)	**0.0076**	102	0.686 (0.586-0.774)	**0.0001**
GVNTs												
APRI score	68	0.583 (0.457-0.702)	0.4108	38	0.559 (0.389-0.720)	0.5561	67	0.575 (0.448-0.695)	0.3073	104	0.612 (0.511-0.706)	0.1463
AAR score	68	0.648 (0.523-0.760)	0.0691	38	0.601 (0.430-0.756)	0.2862	67	0.637 (0.510-0.751)	**0.0499**	104	0.536 (0.436-0.635)	0.6585
FIB4 score	68	0.598 (0.472-0.715)	0.3457	38	0.478 (0.314-0.646)	0.8230	67	0.614 (0.487-0.731)	0.1072	104	0.646 (0.546-0.738)	**0.0480**
King score	66	0.558 (0.431-0.680)	0.5559	38	0.513 (0.346-0.678)	0.8954	67	0.616 (0.489-0.732)	0.1092	102	0.627 (0.525-0.721)	0.1144
Lok score	66	0.626 (0.498-0.742)	0.1504	38	0.536 (0.367-0.699)	0.7158	67	0.497 (0.372-0.622)	0.9661	102	0.524 (0.422-0.624)	0.7894
PSR^∗^	67	0.631 (0.505-0.746)	0.2201	37	0.615 (0.441-0.770)	0.2333	60	0.555 (0.421-0.684)	0.4711	81	0.579 (0.464-0.687)	0.4057
Contrast-enhanced CT	64	0.731 (0.605-0.834)	**0.0316**	35	0.639 (0.460-0.794)	0.1502	64	0.602 (0.472-0.723)	0.1628	100	0.661 (0.559-0.753)	**0.0259**

^∗^PSR was not available in patients with splenectomy. APRI: aspartate aminotransferase to platelet ratio index; AAR: aspartate aminotransferase to alanine aminotransferase ratio; FIB4: fibrosis 4 index; PSR: platelet count to spleen diameter ratio; CT: computed tomography; AUC: area under the curve; CI: confidence interval; EVs: esophageal varices; EVNTs: esophageal varices needing treatment; GVs: gastric varices; GVNTs: gastric varices needing treatment.

## Data Availability

The data used to support the findings of this study are available from the corresponding authors upon request.

## Abbreviations

GEVs: Gastroesophageal varices
EVL: Endoscopic variceal ligation
AST: Aspartate aminotransferase
PLT: Platelet
APRI: Aspartate aminotransferase to platelet ratio index
ALT: Alanine aminotransferase
AAR: Aspartate aminotransferase to alanine aminotransferase ratio
FIB-4: Fibrosis 4 index
CT: Computed tomography
PSR: Platelet count to spleen diameter ratio
EVs: Esophageal varices
GVs: Gastric varices
AUGIB: Acute upper gastrointestinal bleeding
RBC: Red blood cell
Hb: Hemoglobin
WBC: White blood cell
TBIL: Total bilirubin
DBIL: Direct bilirubin
ALB: Albumin
AKP: Alkaline phosphatase
GGT-*γ*: γ-glutamine transferase
BUN: Blood urea nitrogen
SCr: Serum creatinine
PT: Prothrombin time
APTT: Activated partial thromboplastin time
INR: International normalized ratio
MELD: Model for end-stage of liver disease
RC: Red color
EVNTs: Esophageal varices needing treatment
GVNTs: Gastric varices needing treatment
ROC: Receiver operating characteristic
AUC: Area under the curve
NPV: Negative predictive value.

## Supplementary Materials

Supplementary Materials Supplementary Table 1: kappa statistics of diagnosing the presence of esophageal varices and gastric varices on contrast-enhanced CT. Supplementary Table 2: diagnostic performance of noninvasive approaches on the basis of Baveno VI criteria: an overview.
