# Supplementary material for: Contrast-Enhanced CT May Be a Diagnostic Alternative for Gastroesophageal Varices in Cirrhosis with and without Previous Endoscopic Variceal Therapy
Source: Gastroenterol Res Pract. 2019 Oct 20;2019:6704673. doi: 10.1155/2019/6704673 (PMC6855090; doi:10.1155/2019/6704673)
Supplement: Supplementary Materials — Supplementary Table 1: kappa statistics of diagnosing the presence of esophageal varices and gastric varices on contrast-enhanced CT. Supplementary Table 2: diagnostic performance of noninvasive approaches on the basis of Baveno VI criteria: an overview. [file 6704673.f1.docx]

| **Supplementary Table 1. Kappa statistics of diagnosing the presence of esophageal varices and gastric varices on contrast-enhanced CT.** | | | | | | | | |
| --- | --- | --- | --- | --- | --- | --- | --- | --- |
| **Variables** | **Primary prophylaxis  population** | | **Acute bleeding  population** | | **Previous bleeding  population** | | **Secondary prophylaxis  population** | |
|  | **Kappa** | **P value** | **Kappa** | **P value** | **Kappa** | **P value** | **Kappa** | **P value** |
| **EVs** | 0.948 | <0.0001 | 1.000 | <0.0001 | 1.000 | <0.0001 | 1.000 | <0.0001 |
| **GVs** | 0.966 | <0.0001 | 0.888 | <0.0001 | 1.000 | <0.0001 | 0.903 | <0.0001 |
| **Abbreviations:** EVs-Esophageal Varices; GVs-Gastric Varices. | | | | | | | | |

| **Supplementary Table 2. Diagnostic performance of non-invasive approaches on the basis of Baveno VI criteria: an overview** | | | | | | | | |
| --- | --- | --- | --- | --- | --- | --- | --- | --- |
| **First author (Year)** | **Country** | **Target population** | | **Non-invasive approaches** | **Group** | **No Pts.** | **Spared endoscopy (%)** | **Missed EVNTs (%)** |
|  |  | **Inclusion criteria** | **Exclusion criteria** |  |  |  |  |  |
| Jangouk (2016) | USA | cACLD | 1.Previous or present ascites, variceal bleeding, jaundice, or hepatic encephalopathy | Baveno VI criteria | USA | 161 | 26 | 0 |
|  |  |  |  |  | Italy | 101 | 16 | 0 |
|  |  |  |  | Baveno VI criteria+MELD=6 | USA | 161 | 38 | 0 |
|  |  |  |  |  | Italy | 101 | 28 | 0 |
| Maurice (2016) | UK | cACLD | 1.Child Pugh B with ascites, encephalopathy or previous variceal bleeding, and Child-Pugh C 2.Use of non-selective beta blocker | Baveno VI criteria | Endoscopy within 6 months  of TE | 219 | 30 | 3.3 |
|  |  |  |  |  | Endoscopy within 12 months of TE | 310 | 33 | 2 |
| Augustin (2017) | Spain | cACLD | 1.Previous or present ascites, variceal bleeding, jaundice, or hepatic encephalopathy 2.Previous or ongoing treatment for portal hypertension | Baveno VI criteria |  | 925 | 21.5 | 1.5 |
|  |  |  |  |  |  |  |  |  |
|  |  |  |  | Baveno VI criteria+MELD=6 |  | 463 | 21 | 2 |
|  |  |  |  |  |  |  |  |  |
|  |  |  |  | Expanded-Baveno VI criteria |  | 925 | 40 | 1.6 |
|  |  |  |  |  |  |  |  |  |
|  |  |  |  | Expanded-Baveno VI criteria+MELD=6 |  | 883 | 45.8 | 1.7 |
|  |  |  |  |  |  |  |  |  |
| Silva (2017) | Portugal | 1.Cirrhosis 2.Compensated 3.LSM>12.5 kPa | 1.Present ascites and hepatic encephalopathy 2.Previous variceal bleeding 3.Previous endoscopic treatment 4.Child-Pugh B or C 5.Use of non-selective beta blocker | Baveno VI criteria |  | 97 | 11.3 | 0 |
|  |  |  |  |  |  |  |  |  |
|  |  |  |  |  |  |  |  |  |
|  |  |  |  | LSM<30kPa+PLT≥120×10^9^/L |  | 97 | 27.8 | 0 |
|  |  |  |  |  |  |  |  |  |
|  |  |  |  |  |  |  |  |  |
| Bae (2018) | Korea | cACLD | 1.Previous or present ascites, variceal bleeding, jaundice, or hepatic encephalopathy 2.Previous endoscopic treatment | Baveno VI criteria |  | 282 | 27.6 | 3.8 |
|  |  |  |  |  |  |  |  |  |
|  |  |  |  | Expanded-Baveno VI criteria |  | 282 | 51.7 | 6.8 |
|  |  |  |  |  |  |  |  |  |
| Bellan (2018) | Italy | 1.Cirrhosis  2.Hepatitis C | History of alcohol abuse | Baveno VI criteria |  | 160 | 21 | 2.9 |
|  |  |  |  |  |  |  |  |  |
| Calès (2018) | France | Cirrhosis | 1.Previous variceal bleeding 2.Previous endoscopic treatment | VariScreen |  | 211 | 69.2 | 5.6 |
| Colecchia (2018) | Italy | cACLD | 1.Present ascites and hepatic encephalopathy 2.Previous variceal bleeding 3.Previous endoscopic treatment 4.Use non-selective beta blocker | Baveno VI criteria | Internal | 240 | 21.7 | 1.9 |
|  |  |  |  |  | External | 115 | 16.5 | 0 |
|  |  |  |  | SSM≤46kPa | Internal | 240 | 35.8 | 1.2 |
|  |  |  |  |  | External | 115 | 30.4 | 0 |
|  |  |  |  | Baveno VI criteria+SSM≤46kPa | Internal | 240 | 43.8 | 1.9 |
|  |  |  |  |  | External | 115 | 37.4 | 0 |
| Matsui (2018) | Japan | 1.Cirrhosis 2.Compensated | 1.Present ascites and hepatic encephalopathy 2.Previous endoscopic treatment | Baveno VI criteria | Training | 272 | 61.4 | 1.2 |
|  |  |  |  |  | Validation | 112 | 59.8 | 0 |
|  |  |  |  | Modified Baveno VI criteria | Training | 272 | 37.5 | 0.98 |
|  |  |  |  |  | Validation | 112 | 35.7 | 0 |
|  |  |  |  | MRE criteria | Training | 272 | 41.9 | 0.9 |
|  |  |  |  |  | Validation | 243 | 32.1 | 0 |
| Moctezuma-Velazquez (2018) | Canada | 1.cACLD 2.PBC or PSC | 1.Previou ascites, variceal bleeding, jaundice, or hepatic encephalopathy 2.Previous endoscopic treatment 3.Use of non-selective beta blocker | Baveno VI criteria | PBC | 147 | 39 | 0 |
|  |  |  |  |  | PSC | 80 | 30 | 0 |
|  |  |  |  | Expanded-Baveno VI criteria | PBC | 147 | 58 | 6 |
|  |  |  |  |  | PSC | 80 | 45 | 3 |
| Petta (2018) | Italy | 1.Cirrhosis 2.Compensated 3.NAFLD 4.LSM>11.5kPa for M probe and LSM>11kPa for XL probe | 1.Child-Pugh B or C 2.Previous endoscopic treatment | Baveno VI criteria | Training | 314 | 33.8 | 0.9 |
|  |  |  |  |  | Validation | 338 | 33.4 | 4.4 |
|  |  |  |  | Expanded-Baveno VI criteria | Training | 314 | 58 | 3.8 |
|  |  |  |  |  | Validation | 338 | 54.1 | 4.4 |
|  |  |  |  | PLT>110×10^9^/L+LSM<30kPa for M probe | Training | 314 | 68.5 | 4.2 |
|  |  |  |  |  | Validation | 338 | 61.8 | 4.8 |
|  |  |  |  | PLT>110×10^9^/L+ LSM<2kPa for XL probe | Training | 314 | 65 | 4.9 |
|  |  |  |  |  | Validation | 138 | 46.4 | 1.6 |
| **Notes:** Baveno VI criteria: PLT>150×10^9^/L+LSM<20kPa. Expanded Baveno VI criteria: PLT>110×10^9^/L+LSM<25kPa. Modified Baveno VI criteria: PLT>180×10^9^/L+LSM<11.5kPa. MRE criteria: PLT>180×10^9^/L+MRE-LSM<4.2kPa. **Abbreviations:**  EVNTs-Esophageal Varices Needing Treatment; cACLD-compensated Advanced Chronic Liver Disease; TE-Transient Elastography; VCTE-Vibration-Controlled Transient Elastography; MRE-Magnetic Resonance Elastography; LSM-Liver Stiffness Measurement; PLT-Platelet; MELD-Model for End-stage Liver Disease; HCV- Hepatitis C Virus; NAFLD-Non-Alcoholic Fatty Liver Disease; PBC-Primary Biliary Cholangitis; PSC-Primary Sclerosing Cholangitis. | | | | | | | | |
